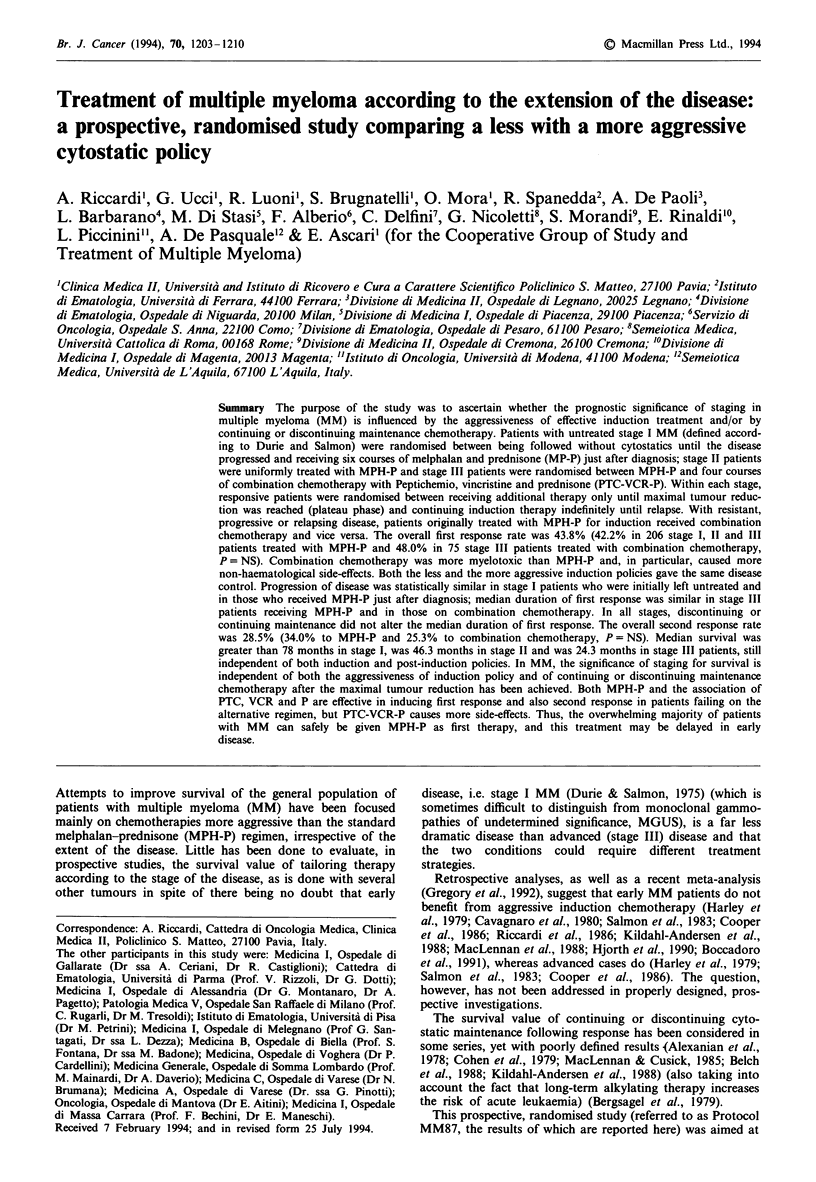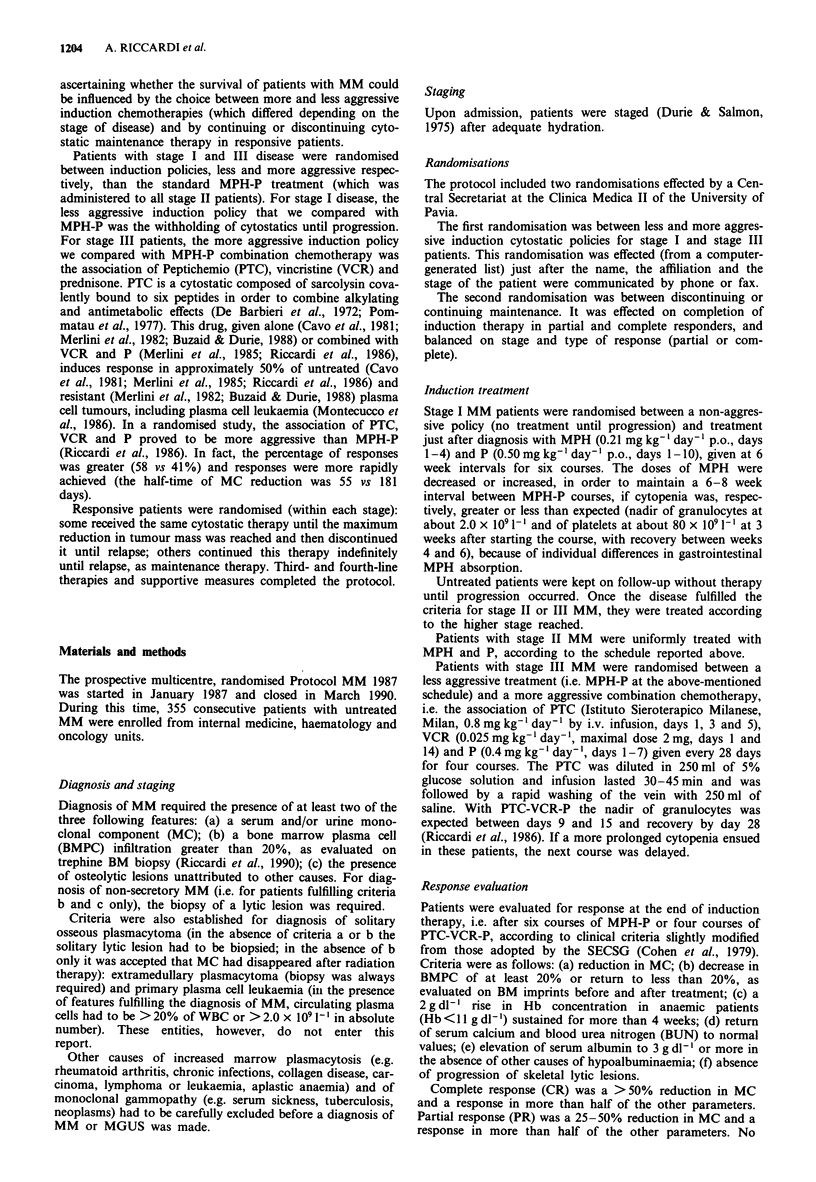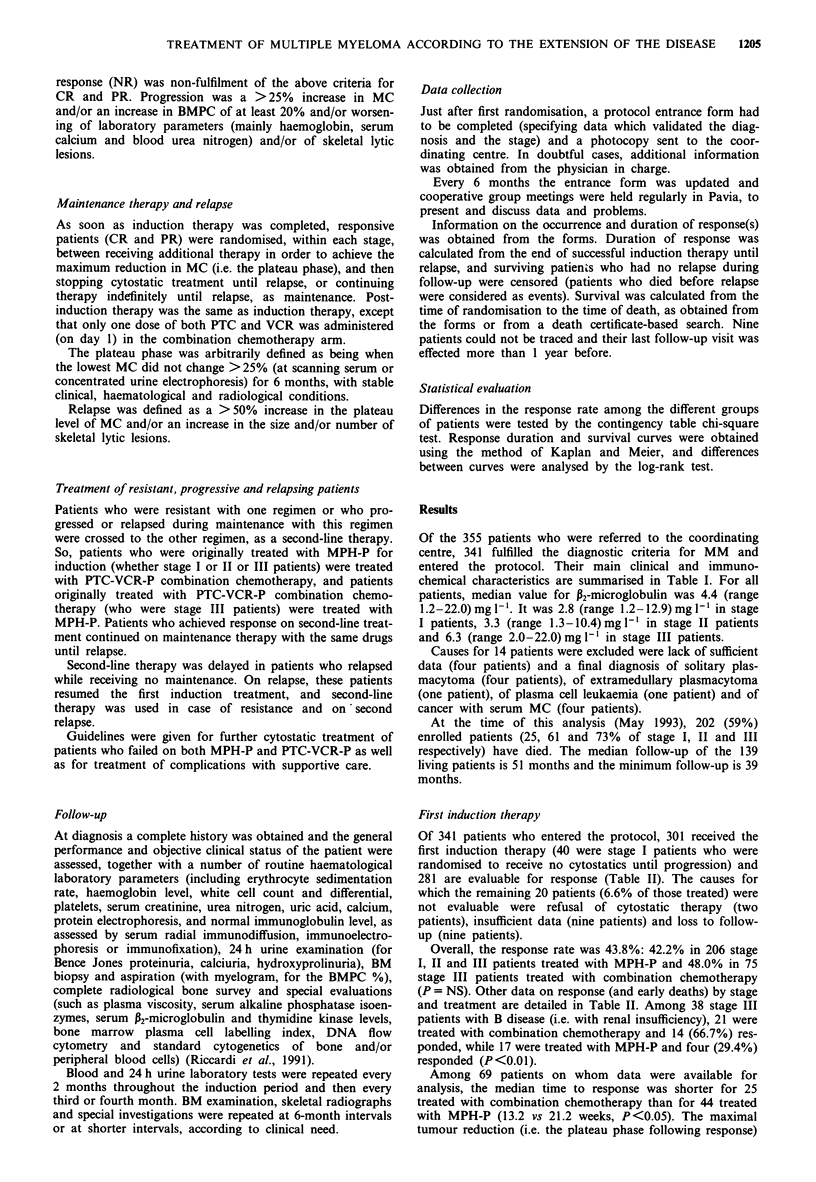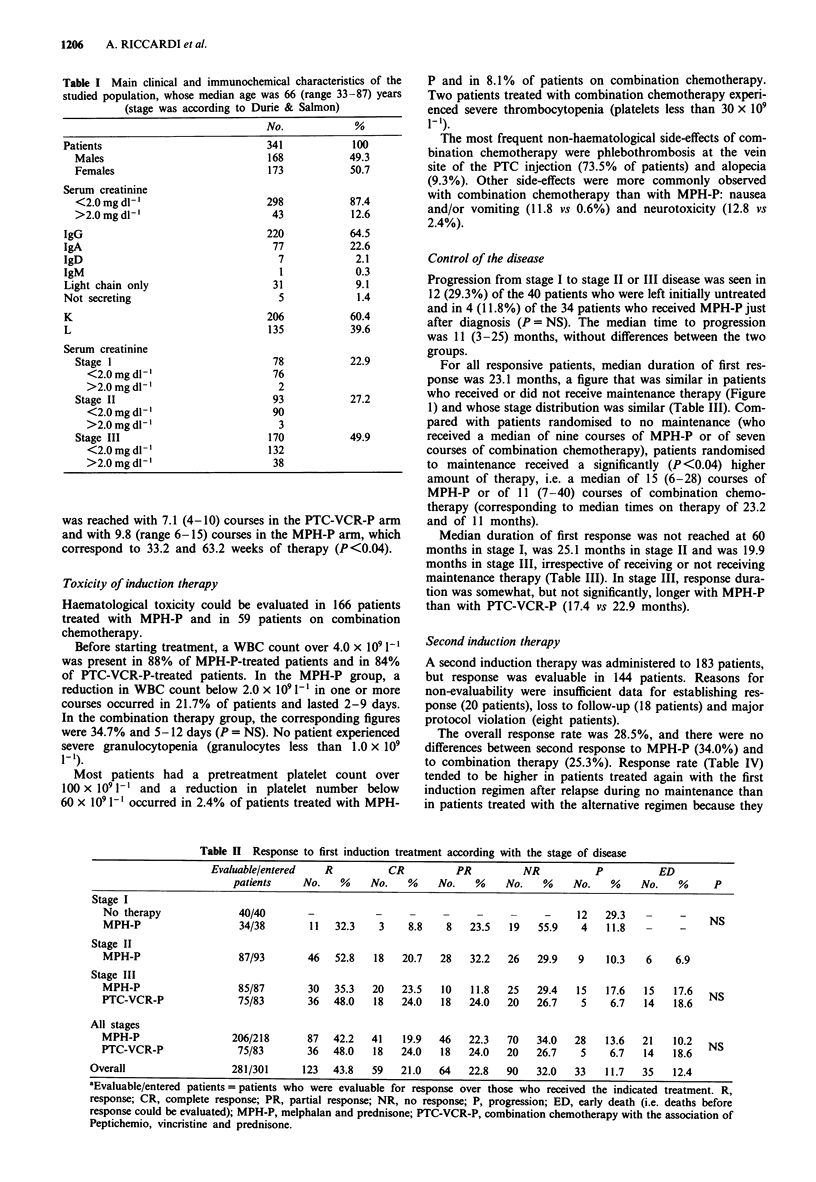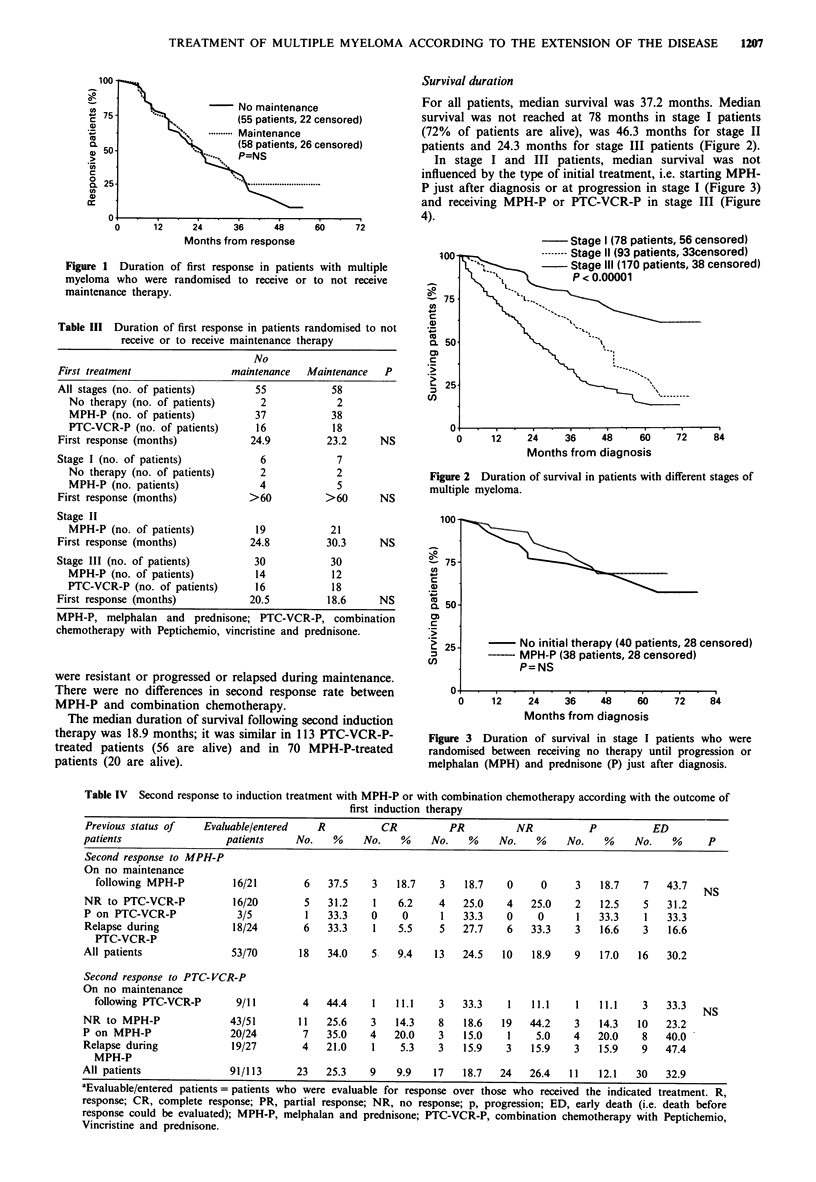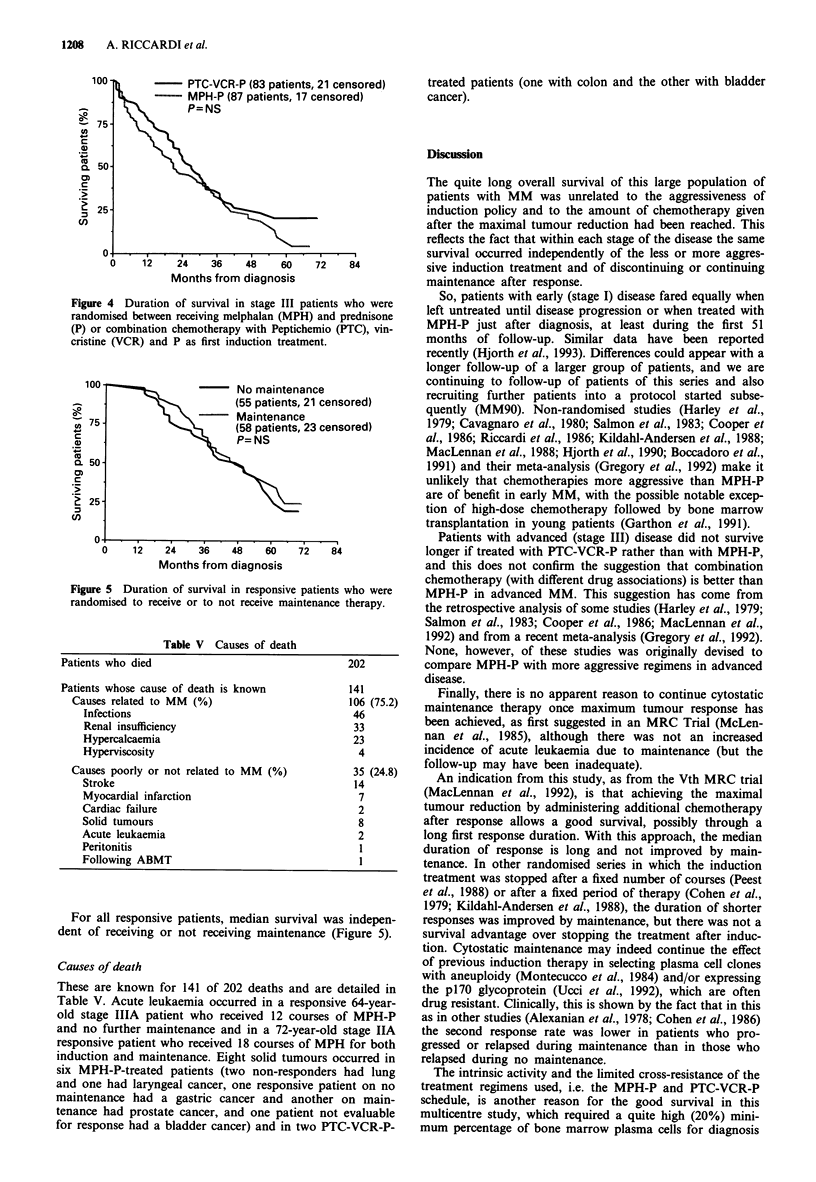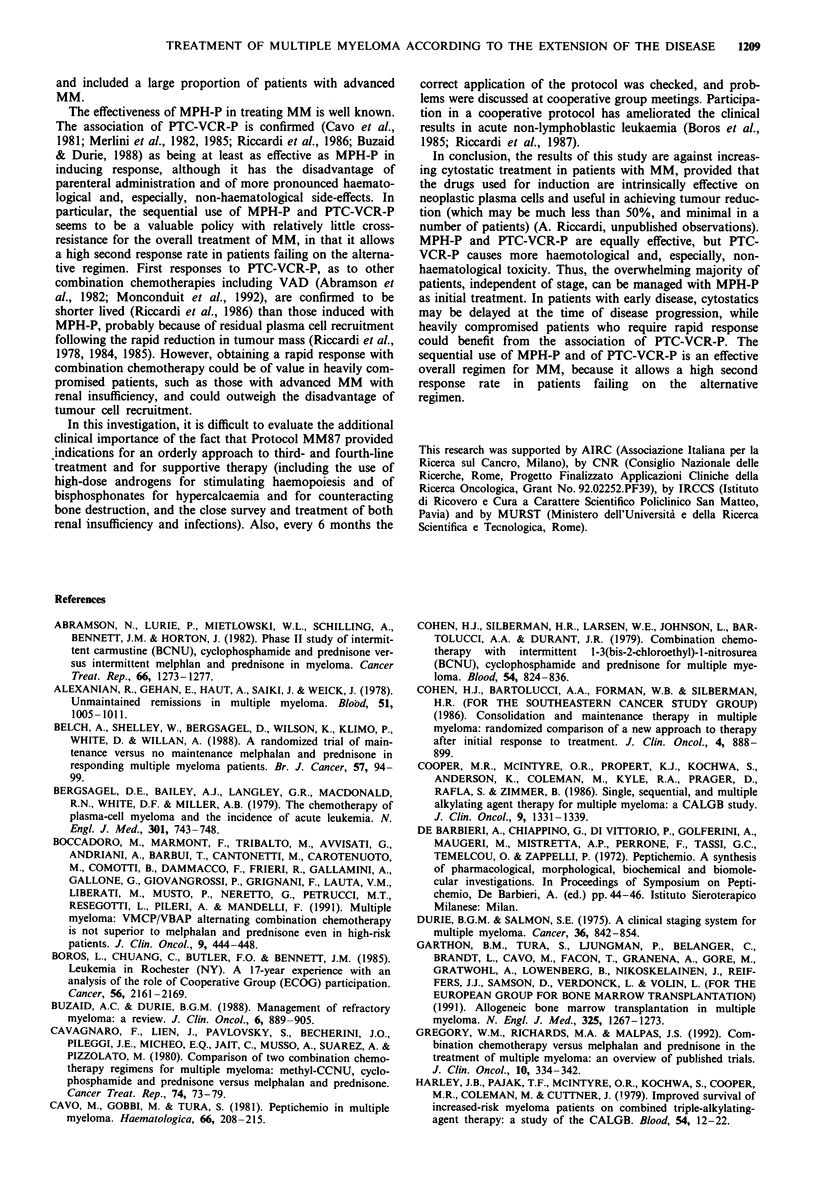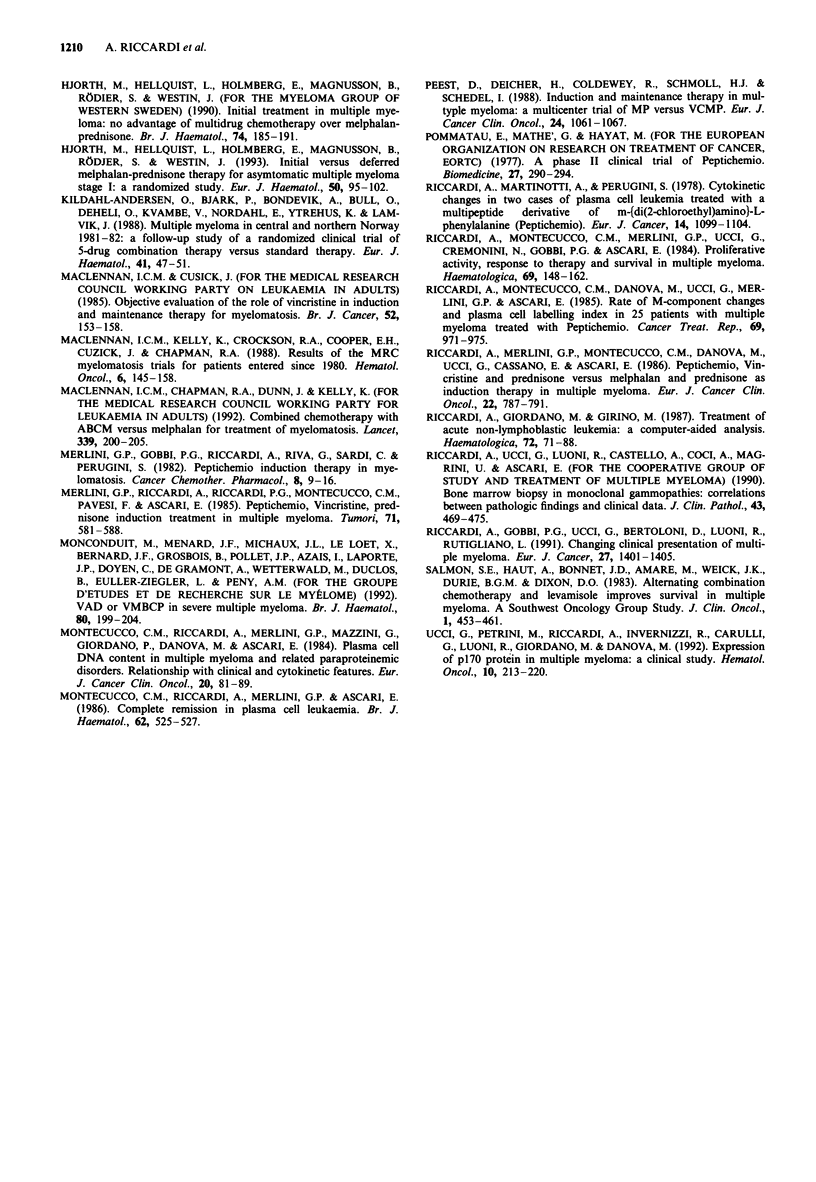# Treatment of multiple myeloma according to the extension of the disease: a prospective, randomised study comparing a less with a more aggressive cystostatic policy. Cooperative Group of Study and Treatment of Multiple Myeloma.

**DOI:** 10.1038/bjc.1994.474

**Published:** 1994-12

**Authors:** A. Riccardi, G. Ucci, R. Luoni, S. Brugnatelli, O. Mora, R. Spanedda, A. De Paoli, L. Barbarano, M. Di Stasi, F. Alberio

**Affiliations:** Clinica Medica II, Università and Istituto di Ricovero e Cura a Carattere Scientifico Policlinico S. Matteo, Pavia, Italy.

## Abstract

The purpose of the study was to ascertain whether the prognostic significance of staging in multiple myeloma (MM) is influenced by the aggressiveness of effective induction treatment and/or by continuing or discontinuing maintenance chemotherapy. Patients with untreated stage I MM (defined according to Durie and Salmon) were randomised between being followed without cytostatics until the disease progressed and receiving six courses of melphalan and prednisone (MP-P) just after diagnosis; stage II patients were uniformly treated with MPH-P and stage III patients were randomised between MPH-P and four courses of combination chemotherapy with Peptichemio, vincristine and prednisone (PTC-VCR-P). Within each stage, responsive patients were randomised between receiving additional therapy only until maximal tumour reduction was reached (plateau phase) and continuing induction therapy indefinitely until relapse. With resistant, progressive or relapsing disease, patients originally treated with MPH-P for induction received combination chemotherapy and vice versa. The overall first response rate was 43.8% (42.2% in 206 stage I, II and III patients treated with MPH-P and 48.0% in 75 stage III patients treated with combination chemotherapy, P = NS). Combination chemotherapy was more myelotoxic than MPH-P and, in particular, caused more non-haematological side-effects. Both the less and the more aggressive induction policies gave the same disease control. Progression of disease was statistically similar in stage I patients who were initially left untreated and in t hose who received MPH-P just after diagnosis; median duration of first response was similar in stage III patients receiving MPH-P and in those on combination chemotherapy. In all stages, discontinuing or continuing maintenance did not alter the median duration of first response. The overall second response rate was 28.5% (34.0% to MPH-P and 25.3% to combination chemotherapy, P = NS). Median survival was greater than 78 months in stage I, was 46.3 months in stage II and was 24.3 months in stage III patients, still independent of both induction and post-induction policies. In MM, the significance of staging for survival is independent of both the aggressiveness of induction and of continuing or discontinuing maintenance chemotherapy after the maximal tumor reduction has been achieved. Both MPH-P and and the association of PTC, VCR and P are effective in inducing first response and also second response in patients failing on the alternative regimen, but PTC-VCR-P causes more side effects. Thus, the overwhelming majority of patients with MM can safely be given MPH-P as first therapy, and this treatment may be delayed in early diseases.


Br. J. Cancer (1994), 70, 1203–1210 © Macmillan Press Ltd., 1994

### Treatment of multiple myeloma according to the extension of the disease: a prospective, randomised study comparing a less with a more aggressive cytostatic policy

A. Riccardi[1], G. Ucci[1], R. Luoni[1], S. Brugnatelli[1], O. Mora[1], R. Spanedda[2], A. De Paoli[3], L. Barbarano[4], M. Di Stasi[5], F. Alberio[6], C. Delfini[7], G. Nicoletti[8], S. Morandi[9], E. Rinaldi[10], L. Piccinini[11], A. De Pasquale[12] & E. Ascari[1] (for the Cooperative Group of Study and Treatment of Multiple Myeloma)

[1]*Clinica Medica II, Università and Istituto di Ricovero e Cura a Carattere Scientifico Policlinico S. Matteo, 27100 Pavia;* [2]*Istituto di Ematologia, Università di Ferrara, 44100 Ferrara;* [3]*Divisione di Medicina II, Ospedale di Legnano, 20025 Legnano;* [4]*Divisione di Ematologia, Ospedale di Niguarda, 20100 Milan,* [5]*Divisione di Medicina I, Ospedale di Piacenza, 29100 Piacenza;* [6]*Servizio di Oncologia, Ospedale S. Anna, 22100 Como;* [7]*Divisione di Ematologia, Ospedale di Pesaro, 61100 Pesaro;* [8]*Semeiotica Medica, Università Cattolica di Roma, 00168 Rome;* [9]*Divisione di Medicina II, Ospedale di Cremona, 26100 Cremona;* [10]*Divisione di Medicina I, Ospedale di Magenta, 20013 Magenta;* [11]*Istituto di Oncologia, Università di Modena, 41100 Modena;* [12]*Semeiotica Medica, Università de L'Aquila, 67100 L'Aquila, Italy.*

**Summary** The purpose of the study was to ascertain whether the prognostic significance of staging in multiple myeloma (MM) is influenced by the aggressiveness of effective induction treatment and/or by continuing or discontinuing maintenance chemotherapy. Patients with untreated stage I MM (defined according to Durie and Salmon) were randomised between being followed without cytostatics until the disease progressed and receiving six courses of melphalan and prednisone (MP-P) just after diagnosis; stage II patients were uniformly treated with MPH-P and stage III patients were randomised between MPH-P and four courses of combination chemotherapy with Peptichemio, vincristine and prednisone (PTC-VCR-P). Within each stage, responsive patients were randomised between receiving additional therapy only until maximal tumour reduction was reached (plateau phase) and continuing induction therapy indefinitely until relapse. With resistant, progressive or relapsing disease, patients originally treated with MPH-P for induction received combination chemotherapy and vice versa. The overall first response rate was 43.8% (42.2% in 206 stage I, II and III patients treated with MPH-P and 48.0% in 75 stage III patients treated with combination chemotherapy, $P$ = NS). Combination chemotherapy was more myelotoxic than MPH-P and, in particular, caused more non-haematological side-effects. Both the less and the more aggressive induction policies gave the same disease control. Progression of disease was statistically similar in stage I patients who were initially left untreated and in those who received MPH-P just after diagnosis; median duration of first response was similar in stage III patients receiving MPH-P and in those on combination chemotherapy. In all stages, discontinuing or continuing maintenance did not alter the median duration of first response. The overall second response rate was 28.5% (34.0% to MPH-P and 25.3% to combination chemotherapy, $P$ = NS). Median survival was greater than 78 months in stage I, was 46.3 months in stage II and was 24.3 months in stage III patients, still independent of both induction and post-induction policies. In MM, the significance of staging for survival is independent of both the aggressiveness of induction policy and of continuing or discontinuing maintenance chemotherapy after the maximal tumour reduction has been achieved. Both MPH-P and the association of PTC, VCR and P are effective in inducing first response and also second response in patients failing on the alternative regimen, but PTC-VCR-P causes more side-effects. Thus, the overwhelming majority of patients with MM can safely be given MPH-P as first therapy, and this treatment may be delayed in early disease.

Attempts to improve survival of the general population of patients with multiple myeloma (MM) have been focused mainly on chemotherapies more aggressive than the standard melphalan–prednisone (MPH-P) regimen, irrespective of the extent of the disease. Little has been done to evaluate, in prospective studies, the survival value of tailoring therapy according to the stage of the disease, as is done with several other tumours in spite of there being no doubt that early disease, i.e. stage I MM (Durie & Salmon, 1975) (which is sometimes difficult to distinguish from monoclonal gammopathies of undetermined significance, MGUS), is a far less dramatic disease than advanced (stage III) disease and that the two conditions could require different treatment strategies.

Retrospective analyses, as well as a recent meta-analysis (Gregory *et al.*, 1992), suggest that early MM patients do not benefit from aggressive induction chemotherapy (Harley *et al.*, 1979; Cavagnaro *et al.*, 1980; Salmon *et al.*, 1983; Cooper *et al.*, 1986; Riccardi *et al.*, 1986; Kildahl-Andersen *et al.*, 1988; MacLennan *et al.*, 1988; Hjorth *et al.*, 1990; Boccadoro *et al.*, 1991), whereas advanced cases do (Harley *et al.*, 1979; Salmon *et al.*, 1983; Cooper *et al.*, 1986). The question, however, has not been addressed in properly designed, prospective investigations.

The survival value of continuing or discontinuing cytostatic maintenance following response has been considered in some series, yet with poorly defined results (Alexanian *et al.*, 1978; Cohen *et al.*, 1979; MacLennan & Cusick, 1985; Belch *et al.*, 1988; Kildahl-Andersen *et al.*, 1988) (also taking into account the fact that long-term alkylating therapy increases the risk of acute leukaemia) (Bergsagel *et al.*, 1979).

This prospective, randomised study (referred to as Protocol MM87, the results of which are reported here) was aimed at

Correspondence: A. Riccardi, Cattedra di Oncologia Medica, Clinica Medica II, Policlinico S. Matteo, 27100 Pavia, Italy.

The other participants in this study were: Medicina I, Ospedale di Gallarate (Dr ssa A. Ceriani, Dr R. Castiglioni); Cattedra di Ematologia, Università di Parma (Prof. V. Rizzoli, Dr G. Dotti); Medicina I, Ospedale di Alessandria (Dr G. Montanaro, Dr A. Pagetto); Patologia Medica V, Ospedale San Raffaele di Milano (Prof. C. Rugarli, Dr M. Tresoldi); Istituto di Ematologia, Università di Pisa (Dr M. Petrini); Medicina I, Ospedale di Melegnano (Prof G. Santagati, Dr ssa L. Dezza); Medicina B, Ospedale di Biella (Prof. S. Fontana, Dr ssa M. Badone); Medicina, Ospedale di Voghera (Dr P. Cardellini); Medicina Generale, Ospedale di Somma Lombardo (Prof. M. Mainardi, Dr A. Daverio); Medicina C, Ospedale di Varese (Dr N. Brumana); Medicina A, Ospedale di Varese (Dr. ssa G. Pinotti); Oncologia, Ospedale di Mantova (Dr E. Aitini); Medicina I, Ospedale di Massa Carrara (Prof. F. Bechini, Dr E. Maneschi).

Received 7 February 1994; and in revised form 25 July 1994.

ascertaining whether the survival of patients with MM could be influenced by the choice between more and less aggressive induction chemotherapies (which differed depending on the stage of disease) and by continuing or discontinuing cytostatic maintenance therapy in responsive patients.

Patients with stage I and III disease were randomised between induction policies, less and more aggressive respectively, than the standard MPH-P treatment (which was administered to all stage II patients). For stage I disease, the less aggressive induction policy that we compared with MPH-P was the withholding of cytostatics until progression. For stage III patients, the more aggressive induction policy we compared with MPH-P combination chemotherapy was the association of Peptichemio (PTC), vincristine (VCR) and prednisone. PTC is a cytostatic composed of sarcolysin covalently bound to six peptides in order to combine alkylating and antimetabolic effects (De Barbieri *et al.*, 1972; Pommatau *et al.*, 1977). This drug, given alone (Cavo *et al.*, 1981; Merlini *et al.*, 1982; Buzaid & Durie, 1988) or combined with VCR and P (Merlini *et al.*, 1985; Riccardi *et al.*, 1986), induces response in approximately 50% of untreated (Cavo *et al.*, 1981; Merlini *et al.*, 1985; Riccardi *et al.*, 1986) and resistant (Merlini *et al.*, 1982; Buzaid & Durie, 1988) plasma cell tumours, including plasma cell leukaemia (Montecucco *et al.*, 1986). In a randomised study, the association of PTC, VCR and P proved to be more aggressive than MPH-P (Riccardi *et al.*, 1986). In fact, the percentage of responses was greater (58 *vs* 41%) and responses were more rapidly achieved (the half-time of MC reduction was 55 *vs* 181 days).

Responsive patients were randomised (within each stage): some received the same cytostatic therapy until the maximum reduction in tumour mass was reached and then discontinued it until relapse; others continued this therapy indefinitely until relapse, as maintenance therapy. Third- and fourth-line therapies and supportive measures completed the protocol.

### Materials and methods

The prospective multicentre, randomised Protocol MM 1987 was started in January 1987 and closed in March 1990. During this time, 355 consecutive patients with untreated MM were enrolled from internal medicine, haematology and oncology units.

### *Diagnosis and staging*

Diagnosis of MM required the presence of at least two of the three following features: (a) a serum and/or urine monoclonal component (MC); (b) a bone marrow plasma cell (BMPC) infiltration greater than 20%, as evaluated on trephine BM biopsy (Riccardi *et al.*, 1990); (c) the presence of osteolytic lesions unattributed to other causes. For diagnosis of non-secretory MM (i.e. for patients fulfilling criteria b and c only), the biopsy of a lytic lesion was required.

Criteria were also established for diagnosis of solitary osseous plasmacytoma (in the absence of criteria a or b the solitary lytic lesion had to be biopsied; in the absence of b only it was accepted that MC had disappeared after radiation therapy): extramedullary plasmacytoma (biopsy was always required) and primary plasma cell leukaemia (in the presence of features fulfilling the diagnosis of MM, circulating plasma cells had to be $>20\%$ of WBC or $>2.0\times10^{9}\,l^{-1}$ in absolute number). These entities, however, do not enter this report.

Other causes of increased marrow plasmacytosis (e.g. rheumatoid arthritis, chronic infections, collagen disease, carcinoma, lymphoma or leukaemia, aplastic anaemia) and of monoclonal gammopathy (e.g. serum sickness, tuberculosis, neoplasms) had to be carefully excluded before a diagnosis of MM or MGUS was made.

### *Staging*

Upon admission, patients were staged (Durie & Salmon, 1975) after adequate hydration.

### *Randomisations*

The protocol included two randomisations effected by a Central Secretariat at the Clinica Medica II of the University of Pavia.

The first randomisation was between less and more aggressive induction cytostatic policies for stage I and stage III patients. This randomisation was effected (from a computer-generated list) just after the name, the affiliation and the stage of the patient were communicated by phone or fax.

The second randomisation was between discontinuing or continuing maintenance. It was effected on completion of induction therapy in partial and complete responders, and balanced on stage and type of response (partial or complete).

### *Induction treatment*

Stage I MM patients were randomised between a non-aggressive policy (no treatment until progression) and treatment just after diagnosis with MPH ($0.21\,mg\,kg^{-1}\,day^{-1}$ p.o., days 1–4) and P ($0.50\,mg\,kg^{-1}\,day^{-1}$ p.o., days 1–10), given at 6 week intervals for six courses. The doses of MPH were decreased or increased, in order to maintain a 6–8 week interval between MPH-P courses, if cytopenia was, respectively, greater or less than expected (nadir of granulocytes at about $2.0\times10^{9}\,l^{-1}$ and of platelets at about $80\times10^{9}\,l^{-1}$ at 3 weeks after starting the course, with recovery between weeks 4 and 6), because of individual differences in gastrointestinal MPH absorption.

Untreated patients were kept on follow-up without therapy until progression occurred. Once the disease fulfilled the criteria for stage II or III MM, they were treated according to the higher stage reached.

Patients with stage II MM were uniformly treated with MPH and P, according to the schedule reported above.

Patients with stage III MM were randomised between a less aggressive treatment (i.e. MPH-P at the above-mentioned schedule) and a more aggressive combination chemotherapy, i.e. the association of PTC (Istituto Sieroterapico Milanese, Milan, $0.8\,mg\,kg^{-1}\,day^{-1}$ by i.v. infusion, days 1, 3 and 5), VCR ($0.025\,mg\,kg^{-1}\,day^{-1}$, maximal dose 2 mg, days 1 and 14) and P ($0.4\,mg\,kg^{-1}\,day^{-1}$, days 1–7) given every 28 days for four courses. The PTC was diluted in 250 ml of 5% glucose solution and infusion lasted 30–45 min and was followed by a rapid washing of the vein with 250 ml of saline. With PTC-VCR-P the nadir of granulocytes was expected between days 9 and 15 and recovery by day 28 (Riccardi *et al.*, 1986). If a more prolonged cytopenia ensued in these patients, the next course was delayed.

### *Response evaluation*

Patients were evaluated for response at the end of induction therapy, i.e. after six courses of MPH-P or four courses of PTC-VCR-P, according to clinical criteria slightly modified from those adopted by the SECSG (Cohen *et al.*, 1979). Criteria were as follows: (a) reduction in MC; (b) decrease in BMPC of at least 20% or return to less than 20%, as evaluated on BM imprints before and after treatment; (c) a $2\,g\,dl^{-1}$ rise in Hb concentration in anaemic patients ($Hb<11\,g\,dl^{-1}$) sustained for more than 4 weeks; (d) return of serum calcium and blood urea nitrogen (BUN) to normal values; (e) elevation of serum albumin to $3\,g\,dl^{-1}$ or more in the absence of other causes of hypoalbuminaemia; (f) absence of progression of skeletal lytic lesions.

Complete response (CR) was a $>50\%$ reduction in MC and a response in more than half of the other parameters. Partial response (PR) was a 25–50% reduction in MC and a response in more than half of the other parameters. No response (NR) was non-fulfilment of the above criteria for CR and PR. Progression was a >25% increase in MC and/or an increase in BMPC of at least 20% and/or worsening of laboratory parameters (mainly haemoglobin, serum calcium and blood urea nitrogen) and/or of skeletal lytic lesions.

*Maintenance therapy and relapse*

As soon as induction therapy was completed, responsive patients (CR and PR) were randomised, within each stage, between receiving additional therapy in order to achieve the maximum reduction in MC (i.e. the plateau phase), and then stopping cytostatic treatment until relapse, or continuing therapy indefinitely until relapse, as maintenance. Post-induction therapy was the same as induction therapy, except that only one dose of both PTC and VCR was administered (on day 1) in the combination chemotherapy arm.

The plateau phase was arbitrarily defined as being when the lowest MC did not change >25% (at scanning serum or concentrated urine electrophoresis) for 6 months, with stable clinical, haematological and radiological conditions.

Relapse was defined as a >50% increase in the plateau level of MC and/or an increase in the size and/or number of skeletal lytic lesions.

*Treatment of resistant, progressive and relapsing patients*

Patients who were resistant with one regimen or who progressed or relapsed during maintenance with this regimen were crossed to the other regimen, as a second-line therapy. So, patients who were originally treated with MPH-P for induction (whether stage I or II or III patients) were treated with PTC-VCR-P combination chemotherapy, and patients originally treated with PTC-VCR-P combination chemotherapy (who were stage III patients) were treated with MPH-P. Patients who achieved response on second-line treatment continued on maintenance therapy with the same drugs until relapse.

Second-line therapy was delayed in patients who relapsed while receiving no maintenance. On relapse, these patients resumed the first induction treatment, and second-line therapy was used in case of resistance and on second relapse.

Guidelines were given for further cytostatic treatment of patients who failed on both MPH-P and PTC-VCR-P as well as for treatment of complications with supportive care.

*Follow-up*

At diagnosis a complete history was obtained and the general performance and objective clinical status of the patient were assessed, together with a number of routine haematological laboratory parameters (including erythrocyte sedimentation rate, haemoglobin level, white cell count and differential, platelets, serum creatinine, urea nitrogen, uric acid, calcium, protein electrophoresis, and normal immunoglobulin level, as assessed by serum radial immunodiffusion, immunoelectrophoresis or immunofixation), 24 h urine examination (for Bence Jones proteinuria, calciuria, hydroxyprolinuria), BM biopsy and aspiration (with myelogram, for the BMPC %), complete radiological bone survey and special evaluations (such as plasma viscosity, serum alkaline phosphatase isoenzymes, serum $\beta_2$-microglobulin and thymidine kinase levels, bone marrow plasma cell labelling index, DNA flow cytometry and standard cytogenetics of bone and/or peripheral blood cells) (Riccardi *et al.*, 1991).

Blood and 24 h urine laboratory tests were repeated every 2 months throughout the induction period and then every third or fourth month. BM examination, skeletal radiographs and special investigations were repeated at 6-month intervals or at shorter intervals, according to clinical need.

*Data collection*

Just after first randomisation, a protocol entrance form had to be completed (specifying data which validated the diagnosis and the stage) and a photocopy sent to the coordinating centre. In doubtful cases, additional information was obtained from the physician in charge.

Every 6 months the entrance form was updated and cooperative group meetings were held regularly in Pavia, to present and discuss data and problems.

Information on the occurrence and duration of response(s) was obtained from the forms. Duration of response was calculated from the end of successful induction therapy until relapse, and surviving patients who had no relapse during follow-up were censored (patients who died before relapse were considered as events). Survival was calculated from the time of randomisation to the time of death, as obtained from the forms or from a death certificate-based search. Nine patients could not be traced and their last follow-up visit was effected more than 1 year before.

*Statistical evaluation*

Differences in the response rate among the different groups of patients were tested by the contingency table chi-square test. Response duration and survival curves were obtained using the method of Kaplan and Meier, and differences between curves were analysed by the log-rank test.

### Results

Of the 355 patients who were referred to the coordinating centre, 341 fulfilled the diagnostic criteria for MM and entered the protocol. Their main clinical and immunochemical characteristics are summarised in Table I. For all patients, median value for $\beta_2$-microglobulin was 4.4 (range 1.2–22.0) mg $l^{-1}$. It was 2.8 (range 1.2–12.9) mg $l^{-1}$ in stage I patients, 3.3 (range 1.3–10.4) mg $l^{-1}$ in stage II patients and 6.3 (range 2.0–22.0) mg $l^{-1}$ in stage III patients.

Causes for 14 patients were excluded were lack of sufficient data (four patients) and a final diagnosis of solitary plasmacytoma (four patients), of extramedullary plasmacytoma (one patient), of plasma cell leukaemia (one patient) and of cancer with serum MC (four patients).

At the time of this analysis (May 1993), 202 (59%) enrolled patients (25, 61 and 73% of stage I, II and III respectively) have died. The median follow-up of the 139 living patients is 51 months and the minimum follow-up is 39 months.

*First induction therapy*

Of 341 patients who entered the protocol, 301 received the first induction therapy (40 were stage I patients who were randomised to receive no cytostatics until progression) and 281 are evaluable for response (Table II). The causes for which the remaining 20 patients (6.6% of those treated) were not evaluable were refusal of cytostatic therapy (two patients), insufficient data (nine patients) and loss to follow-up (nine patients).

Overall, the response rate was 43.8%: 42.2% in 206 stage I, II and III patients treated with MPH-P and 48.0% in 75 stage III patients treated with combination chemotherapy ($P$ = NS). Other data on response (and early deaths) by stage and treatment are detailed in Table II. Among 38 stage III patients with B disease (i.e. with renal insufficiency), 21 were treated with combination chemotherapy and 14 (66.7%) responded, while 17 were treated with MPH-P and four (29.4%) responded ($P<0.01$).

Among 69 patients on whom data were available for analysis, the median time to response was shorter for 25 treated with combination chemotherapy than for 44 treated with MPH-P (13.2 *vs* 21.2 weeks, $P<0.05$). The maximal tumour reduction (i.e. the plateau phase following response)

**Table I** Main clinical and immunochemical characteristics of the studied population, whose median age was 66 (range 33–87) years (stage was according to Durie & Salmon)

| | No. | % |
|---|---|---|
| Patients | 341 | 100 |
| Males | 168 | 49.3 |
| Females | 173 | 50.7 |
| Serum creatinine | | |
| <2.0 mg dl$^{-1}$ | 298 | 87.4 |
| >2.0 mg dl$^{-1}$ | 43 | 12.6 |
| IgG | 220 | 64.5 |
| IgA | 77 | 22.6 |
| IgD | 7 | 2.1 |
| IgM | 1 | 0.3 |
| Light chain only | 31 | 9.1 |
| Not secreting | 5 | 1.4 |
| K | 206 | 60.4 |
| L | 135 | 39.6 |
| Serum creatinine | | |
| Stage I | 78 | 22.9 |
| <2.0 mg dl$^{-1}$ | 76 | |
| >2.0 mg dl$^{-1}$ | 2 | |
| Stage II | 93 | 27.2 |
| <2.0 mg dl$^{-1}$ | 90 | |
| >2.0 mg dl$^{-1}$ | 3 | |
| Stage III | 170 | 49.9 |
| <2.0 mg dl$^{-1}$ | 132 | |
| >2.0 mg dl$^{-1}$ | 38 | |

was reached with 7.1 (4–10) courses in the PTC-VCR-P arm and with 9.8 (range 6–15) courses in the MPH-P arm, which correspond to 33.2 and 63.2 weeks of therapy ($P<0.04$).

*Toxicity of induction therapy*

Haematological toxicity could be evaluated in 166 patients treated with MPH-P and in 59 patients on combination chemotherapy.

Before starting treatment, a WBC count over $4.0 \times 10^9\,l^{-1}$ was present in 88% of MPH-P-treated patients and in 84% of PTC-VCR-P-treated patients. In the MPH-P group, a reduction in WBC count below $2.0 \times 10^9\,l^{-1}$ in one or more courses occurred in 21.7% of patients and lasted 2–9 days. In the combination therapy group, the corresponding figures were 34.7% and 5–12 days ($P$ = NS). No patient experienced severe granulocytopenia (granulocytes less than $1.0 \times 10^9\,l^{-1}$).

Most patients had a pretreatment platelet count over $100 \times 10^9\,l^{-1}$ and a reduction in platelet number below $60 \times 10^9\,l^{-1}$ occurred in 2.4% of patients treated with MPH-P and in 8.1% of patients on combination chemotherapy. Two patients treated with combination chemotherapy experienced severe thrombocytopenia (platelets less than $30 \times 10^9\,l^{-1}$).

The most frequent non-haematological side-effects of combination chemotherapy were phlebothrombosis at the vein site of the PTC injection (73.5% of patients) and alopecia (9.3%). Other side-effects were more commonly observed with combination chemotherapy than with MPH-P: nausea and/or vomiting (11.8 *vs* 0.6%) and neurotoxicity (12.8 *vs* 2.4%).

*Control of the disease*

Progression from stage I to stage II or III disease was seen in 12 (29.3%) of the 40 patients who were left initially untreated and in 4 (11.8%) of the 34 patients who received MPH-P just after diagnosis ($P$ = NS). The median time to progression was 11 (3–25) months, without differences between the two groups.

For all responsive patients, median duration of first response was 23.1 months, a figure that was similar in patients who received or did not receive maintenance therapy (Figure 1) and whose stage distribution was similar (Table III). Compared with patients randomised to no maintenance (who received a median of nine courses of MPH-P or of seven courses of combination chemotherapy), patients randomised to maintenance received a significantly ($P<0.04$) higher amount of therapy, i.e. a median of 15 (6–28) courses of MPH-P or of 11 (7–40) courses of combination chemotherapy (corresponding to median times on therapy of 23.2 and of 11 months).

Median duration of first response was not reached at 60 months in stage I, was 25.1 months in stage II and was 19.9 months in stage III, irrespective of receiving or not receiving maintenance therapy (Table III). In stage III, response duration was somewhat, but not significantly, longer with MPH-P than with PTC-VCR-P (17.4 *vs* 22.9 months).

*Second induction therapy*

A second induction therapy was administered to 183 patients, but response was evaluable in 144 patients. Reasons for non-evaluability were insufficient data for establishing response (20 patients), loss to follow-up (18 patients) and major protocol violation (eight patients).

The overall response rate was 28.5%, and there were no differences between second response to MPH-P (34.0%) and to combination therapy (25.3%). Response rate (Table IV) tended to be higher in patients treated again with the first induction regimen after relapse during no maintenance than in patients treated with the alternative regimen because they

**Table II** Response to first induction treatment according with the stage of disease

| | Evaluable/entered patients | R No. | R % | CR No. | CR % | PR No. | PR % | NR No. | NR % | P No. | P % | ED No. | ED % | P |
|---|---|---|---|---|---|---|---|---|---|---|---|---|---|---|
| Stage I | | | | | | | | | | | | | | |
| No therapy | 40/40 | – | | – | – | – | – | – | – | 12 | 29.3 | – | – | NS |
| MPH-P | 34/38 | 11 | 32.3 | 3 | 8.8 | 8 | 23.5 | 19 | 55.9 | 4 | 11.8 | – | – | |
| Stage II | | | | | | | | | | | | | | |
| MPH-P | 87/93 | 46 | 52.8 | 18 | 20.7 | 28 | 32.2 | 26 | 29.9 | 9 | 10.3 | 6 | 6.9 | |
| Stage III | | | | | | | | | | | | | | |
| MPH-P | 85/87 | 30 | 35.3 | 20 | 23.5 | 10 | 11.8 | 25 | 29.4 | 15 | 17.6 | 15 | 17.6 | NS |
| PTC-VCR-P | 75/83 | 36 | 48.0 | 18 | 24.0 | 18 | 24.0 | 20 | 26.7 | 5 | 6.7 | 14 | 18.6 | |
| All stages | | | | | | | | | | | | | | |
| MPH-P | 206/218 | 87 | 42.2 | 41 | 19.9 | 46 | 22.3 | 70 | 34.0 | 28 | 13.6 | 21 | 10.2 | NS |
| PTC-VCR-P | 75/83 | 36 | 48.0 | 18 | 24.0 | 18 | 24.0 | 20 | 26.7 | 5 | 6.7 | 14 | 18.6 | |
| Overall | 281/301 | 123 | 43.8 | 59 | 21.0 | 64 | 22.8 | 90 | 32.0 | 33 | 11.7 | 35 | 12.4 | |

[a]Evaluable/entered patients = patients who were evaluable for response over those who received the indicated treatment. R, response; CR, complete response; PR, partial response; NR, no response; P, progression; ED, early death (i.e. deaths before response could be evaluated); MPH-P, melphalan and prednisone; PTC-VCR-P, combination chemotherapy with the association of Peptichemio, vincristine and prednisone.

**Figure 1** Duration of first response in patients with multiple myeloma who were randomised to receive or to not receive maintenance therapy.

**Table III** Duration of first response in patients randomised to not receive or to receive maintenance therapy

| *First treatment* | *No maintenance* | *Maintenance* | *P* |
|---|---|---|---|
| All stages (no. of patients) | 55 | 58 | |
| No therapy (no. of patients) | 2 | 2 | |
| MPH-P (no. of patients) | 37 | 38 | |
| PTC-VCR-P (no. of patients) | 16 | 18 | |
| First response (months) | 24.9 | 23.2 | NS |
| Stage I (no. of patients) | 6 | 7 | |
| No therapy (no. of patients) | 2 | 2 | |
| MPH-P (no. patients) | 4 | 5 | |
| First response (months) | >60 | >60 | NS |
| Stage II | | | |
| MPH-P (no. of patients) | 19 | 21 | |
| First response (months) | 24.8 | 30.3 | NS |
| Stage III (no. of patients) | 30 | 30 | |
| MPH-P (no. of patients) | 14 | 12 | |
| PTC-VCR-P (no. of patients) | 16 | 18 | |
| First response (months) | 20.5 | 18.6 | NS |

MPH-P, melphalan and prednisone; PTC-VCR-P, combination chemotherapy with Peptichemio, vincristine and prednisone.

were resistant or progressed or relapsed during maintenance. There were no differences in second response rate between MPH-P and combination chemotherapy.

The median duration of survival following second induction therapy was 18.9 months; it was similar in 113 PTC-VCR-P-treated patients (56 are alive) and in 70 MPH-P-treated patients (20 are alive).

*Survival duration*

For all patients, median survival was 37.2 months. Median survival was not reached at 78 months in stage I patients (72% of patients are alive), was 46.3 months for stage II patients and 24.3 months for stage III patients (Figure 2).

In stage I and III patients, median survival was not influenced by the type of initial treatment, i.e. starting MPH-P just after diagnosis or at progression in stage I (Figure 3) and receiving MPH-P or PTC-VCR-P in stage III (Figure 4).

**Figure 2** Duration of survival in patients with different stages of multiple myeloma.

**Figure 3** Duration of survival in stage I patients who were randomised between receiving no therapy until progression or melphalan (MPH) and prednisone (P) just after diagnosis.

**Table IV** Second response to induction treatment with MPH-P or with combination chemotherapy according with the outcome of first induction therapy

| *Previous status of patients* | *Evaluable/entered patients* | *R* No. | *R* % | *CR* No. | *CR* % | *PR* No. | *PR* % | *NR* No. | *NR* % | *P* No. | *P* % | *ED* No. | *ED* % | *P* |
|---|---|---|---|---|---|---|---|---|---|---|---|---|---|---|
| *Second response to MPH-P* | | | | | | | | | | | | | | |
| On no maintenance following MPH-P | 16/21 | 6 | 37.5 | 3 | 18.7 | 3 | 18.7 | 0 | 0 | 3 | 18.7 | 7 | 43.7 | NS |
| NR to PTC-VCR-P | 16/20 | 5 | 31.2 | 1 | 6.2 | 4 | 25.0 | 4 | 25.0 | 2 | 12.5 | 5 | 31.2 | |
| P on PTC-VCR-P | 3/5 | 1 | 33.3 | 0 | 0 | 1 | 33.3 | 0 | 0 | 1 | 33.3 | 1 | 33.3 | |
| Relapse during PTC-VCR-P | 18/24 | 6 | 33.3 | 1 | 5.5 | 5 | 27.7 | 6 | 33.3 | 3 | 16.6 | 3 | 16.6 | |
| All patients | 53/70 | 18 | 34.0 | 5 | 9.4 | 13 | 24.5 | 10 | 18.9 | 9 | 17.0 | 16 | 30.2 | |
| *Second response to PTC-VCR-P* | | | | | | | | | | | | | | |
| On no maintenance following PTC-VCR-P | 9/11 | 4 | 44.4 | 1 | 11.1 | 3 | 33.3 | 1 | 11.1 | 1 | 11.1 | 3 | 33.3 | NS |
| NR to MPH-P | 43/51 | 11 | 25.6 | 3 | 14.3 | 8 | 18.6 | 19 | 44.2 | 3 | 14.3 | 10 | 23.2 | |
| P on MPH-P | 20/24 | 7 | 35.0 | 4 | 20.0 | 3 | 15.0 | 1 | 5.0 | 4 | 20.0 | 8 | 40.0 | |
| Relapse during MPH-P | 19/27 | 4 | 21.0 | 1 | 5.3 | 3 | 15.9 | 3 | 15.9 | 3 | 15.9 | 9 | 47.4 | |
| All patients | 91/113 | 23 | 25.3 | 9 | 9.9 | 17 | 18.7 | 24 | 26.4 | 11 | 12.1 | 30 | 32.9 | |

[a]Evaluable/entered patients = patients who were evaluable for response over those who received the indicated treatment. R, response; CR, complete response; PR, partial response; NR, no response; p, progression; ED, early death (i.e. death before response could be evaluated); MPH-P, melphalan and prednisone; PTC-VCR-P, combination chemotherapy with Peptichemio, Vincristine and prednisone.

**Figure 4** Duration of survival in stage III patients who were randomised between receiving melphalan (MPH) and prednisone (P) or combination chemotherapy with Peptichemio (PTC), vincristine (VCR) and P as first induction treatment.

**Figure 5** Duration of survival in responsive patients who were randomised to receive or to not receive maintenance therapy.

**Table V** Causes of death

| | |
|---|---|
| Patients who died | 202 |
| Patients whose cause of death is known | 141 |
| Causes related to MM (%) | 106 (75.2) |
| Infections | 46 |
| Renal insufficiency | 33 |
| Hypercalcaemia | 23 |
| Hyperviscosity | 4 |
| Causes poorly or not related to MM (%) | 35 (24.8) |
| Stroke | 14 |
| Myocardial infarction | 7 |
| Cardiac failure | 2 |
| Solid tumours | 8 |
| Acute leukaemia | 2 |
| Peritonitis | 1 |
| Following ABMT | 1 |

For all responsive patients, median survival was independent of receiving or not receiving maintenance (Figure 5).

### *Causes of death*

These are known for 141 of 202 deaths and are detailed in Table V. Acute leukaemia occurred in a responsive 64-year-old stage IIIA patient who received 12 courses of MPH-P and no further maintenance and in a 72-year-old stage IIA responsive patient who received 18 courses of MPH for both induction and maintenance. Eight solid tumours occurred in six MPH-P-treated patients (two non-responders had lung and one had laryngeal cancer, one responsive patient on no maintenance had a gastric cancer and another on maintenance had prostate cancer, and one patient not evaluable for response had a bladder cancer) and in two PTC-VCR-P-treated patients (one with colon and the other with bladder cancer).

### Discussion

The quite long overall survival of this large population of patients with MM was unrelated to the aggressiveness of induction policy and to the amount of chemotherapy given after the maximal tumour reduction had been reached. This reflects the fact that within each stage of the disease the same survival occurred independently of the less or more aggressive induction treatment and of discontinuing or continuing maintenance after response.

So, patients with early (stage I) disease fared equally when left untreated until disease progression or when treated with MPH-P just after diagnosis, at least during the first 51 months of follow-up. Similar data have been reported recently (Hjorth *et al.*, 1993). Differences could appear with a longer follow-up of a larger group of patients, and we are continuing to follow-up of patients of this series and also recruiting further patients into a protocol started subsequently (MM90). Non-randomised studies (Harley *et al.*, 1979; Cavagnaro *et al.*, 1980; Salmon *et al.*, 1983; Cooper *et al.*, 1986; Riccardi *et al.*, 1986; Kildahl-Andersen *et al.*, 1988; MacLennan *et al.*, 1988; Hjorth *et al.*, 1990; Boccadoro *et al.*, 1991) and their meta-analysis (Gregory *et al.*, 1992) make it unlikely that chemotherapies more aggressive than MPH-P are of benefit in early MM, with the possible notable exception of high-dose chemotherapy followed by bone marrow transplantation in young patients (Garthon *et al.*, 1991).

Patients with advanced (stage III) disease did not survive longer if treated with PTC-VCR-P rather than with MPH-P, and this does not confirm the suggestion that combination chemotherapy (with different drug associations) is better than MPH-P in advanced MM. This suggestion has come from the retrospective analysis of some studies (Harley *et al.*, 1979; Salmon *et al.*, 1983; Cooper *et al.*, 1986; MacLennan *et al.*, 1992) and from a recent meta-analysis (Gregory *et al.*, 1992). None, however, of these studies was originally devised to compare MPH-P with more aggressive regimens in advanced disease.

Finally, there is no apparent reason to continue cytostatic maintenance therapy once maximum tumour response has been achieved, as first suggested in an MRC Trial (McLennan *et al.*, 1985), although there was not an increased incidence of acute leukaemia due to maintenance (but the follow-up may have been inadequate).

An indication from this study, as from the Vth MRC trial (MacLennan *et al.*, 1992), is that achieving the maximal tumour reduction by administering additional chemotherapy after response allows a good survival, possibly through a long first response duration. With this approach, the median duration of response is long and not improved by maintenance. In other randomised series in which the induction treatment was stopped after a fixed number of courses (Peest *et al.*, 1988) or after a fixed period of therapy (Cohen *et al.*, 1979; Kildahl-Andersen *et al.*, 1988), the duration of shorter responses was improved by maintenance, but there was not a survival advantage over stopping the treatment after induction. Cytostatic maintenance may indeed continue the effect of previous induction therapy in selecting plasma cell clones with aneuploidy (Montecucco *et al.*, 1984) and/or expressing the p170 glycoprotein (Ucci *et al.*, 1992), which are often drug resistant. Clinically, this is shown by the fact that in this as in other studies (Alexanian *et al.*, 1978; Cohen *et al.*, 1986) the second response rate was lower in patients who progressed or relapsed during maintenance than in those who relapsed during no maintenance.

The intrinsic activity and the limited cross-resistance of the treatment regimens used, i.e. the MPH-P and PTC-VCR-P schedule, is another reason for the good survival in this multicentre study, which required a quite high (20%) minimum percentage of bone marrow plasma cells for diagnosis and included a large proportion of patients with advanced MM.

The effectiveness of MPH-P in treating MM is well known. The association of PTC-VCR-P is confirmed (Cavo *et al.*, 1981; Merlini *et al.*, 1982, 1985; Riccardi *et al.*, 1986; Buzaid & Durie, 1988) as being at least as effective as MPH-P in inducing response, although it has the disadvantage of parenteral administration and of more pronounced haematological and, especially, non-haematological side-effects. In particular, the sequential use of MPH-P and PTC-VCR-P seems to be a valuable policy with relatively little cross-resistance for the overall treatment of MM, in that it allows a high second response rate in patients failing on the alternative regimen. First responses to PTC-VCR-P, as to other combination chemotherapies including VAD (Abramson *et al.*, 1982; Monconduit *et al.*, 1992), are confirmed to be shorter lived (Riccardi *et al.*, 1986) than those induced with MPH-P, probably because of residual plasma cell recruitment following the rapid reduction in tumour mass (Riccardi *et al.*, 1978, 1984, 1985). However, obtaining a rapid response with combination chemotherapy could be of value in heavily compromised patients, such as those with advanced MM with renal insufficiency, and could outweigh the disadvantage of tumour cell recruitment.

In this investigation, it is difficult to evaluate the additional clinical importance of the fact that Protocol MM87 provided indications for an orderly approach to third- and fourth-line treatment and for supportive therapy (including the use of high-dose androgens for stimulating haemopoiesis and of bisphosphonates for hypercalcaemia and for counteracting bone destruction, and the close survey and treatment of both renal insufficiency and infections). Also, every 6 months the correct application of the protocol was checked, and problems were discussed at cooperative group meetings. Participation in a cooperative protocol has ameliorated the clinical results in acute non-lymphoblastic leukaemia (Boros *et al.*, 1985; Riccardi *et al.*, 1987).

In conclusion, the results of this study are against increasing cytostatic treatment in patients with MM, provided that the drugs used for induction are intrinsically effective on neoplastic plasma cells and useful in achieving tumour reduction (which may be much less than 50%, and minimal in a number of patients) (A. Riccardi, unpublished observations). MPH-P and PTC-VCR-P are equally effective, but PTC-VCR-P causes more haemotological and, especially, non-haematological toxicity. Thus, the overwhelming majority of patients, independent of stage, can be managed with MPH-P as initial treatment. In patients with early disease, cytostatics may be delayed at the time of disease progression, while heavily compromised patients who require rapid response could benefit from the association of PTC-VCR-P. The sequential use of MPH-P and of PTC-VCR-P is an effective overall regimen for MM, because it allows a high second response rate in patients failing on the alternative regimen.

This research was supported by AIRC (Associazione Italiana per la Ricerca sul Cancro, Milano), by CNR (Consiglio Nazionale delle Ricerche, Rome, Progetto Finalizzato Applicazioni Cliniche della Ricerca Oncologica, Grant No. 92.02252.PF39), by IRCCS (Istituto di Ricovero e Cura a Carattere Scientifico Policlinico San Matteo, Pavia) and by MURST (Ministero dell'Università e della Ricerca Scientifica e Tecnologica, Rome).

### References

ABRAMSON, N., LURIE, P., MIETLOWSKI, W.L., SCHILLING, A., BENNETT, J.M. & HORTON, J. (1982). Phase II study of intermittent carmustine (BCNU), cyclophosphamide and prednisone versus intermittent melphlan and prednisone in myeloma. *Cancer Treat. Rep.*, **66**, 1273–1277.

ALEXANIAN, R., GEHAN, E., HAUT, A., SAIKI, J. & WEICK, J. (1978). Unmaintained remissions in multiple myeloma. *Blood*, **51**, 1005–1011.

BELCH, A., SHELLEY, W., BERGSAGEL, D., WILSON, K., KLIMO, P., WHITE, D. & WILLAN, A. (1988). A randomized trial of maintenance versus no maintenance melphalan and prednisone in responding multiple myeloma patients. *Br. J. Cancer*, **57**, 94–99.

BERGSAGEL, D.E., BAILEY, A.J., LANGLEY, G.R., MACDONALD, R.N., WHITE, D.F. & MILLER, A.B. (1979). The chemotherapy of plasma-cell myeloma and the incidence of acute leukemia. *N. Engl. J. Med.*, **301**, 743–748.

BOCCADORO, M., MARMONT, F., TRIBALTO, M., AVVISATI, G., ANDRIANI, A., BARBUI, T., CANTONETTI, M., CAROTENUOTO, M., COMOTTI, B., DAMMACCO, F., FRIERI, R., GALLAMINI, A., GALLONE, G., GIOVANGROSSI, P., GRIGNANI, F., LAUTA, V.M., LIBERATI, M., MUSTO, P., NERETTO, G., PETRUCCI, M.T., RESEGOTTI, L., PILERI, A. & MANDELLI, F. (1991). Multiple myeloma: VMCP/VBAP alternating combination chemotherapy is not superior to melphalan and prednisone even in high-risk patients. *J. Clin. Oncol.*, **9**, 444–448.

BOROS, L., CHUANG, C., BUTLER, F.O. & BENNETT, J.M. (1985). Leukemia in Rochester (NY). A 17-year experience with an analysis of the role of Cooperative Group (ECOG) participation. *Cancer*, **56**, 2161–2169.

BUZAID, A.C. & DURIE, B.G.M. (1988). Management of refractory myeloma: a review. *J. Clin. Oncol.*, **6**, 889–905.

CAVAGNARO, F., LIEN, J., PAVLOVSKY, S., BECHERINI, J.O., PILEGGI, J.E., MICHEO, E.Q., JAIT, C., MUSSO, A., SUAREZ, A. & PIZZOLATO, M. (1980). Comparison of two combination chemotherapy regimens for multiple myeloma: methyl-CCNU, cyclophosphamide and prednisone versus melphalan and prednisone. *Cancer Treat. Rep.*, **74**, 73–79.

CAVO, M., GOBBI, M. & TURA, S. (1981). Peptichemio in multiple myeloma. *Haematologica*, **66**, 208–215.

COHEN, H.J., SILBERMAN, H.R., LARSEN, W.E., JOHNSON, L., BARTOLUCCI, A.A. & DURANT, J.R. (1979). Combination chemotherapy with intermittent 1-3(bis-2-chloroethyl)-1-nitrosurea (BCNU), cyclophosphamide and prednisone for multiple myeloma. *Blood*, **54**, 824–836.

COHEN, H.J., BARTOLUCCI, A.A., FORMAN, W.B. & SILBERMAN, H.R. (FOR THE SOUTHEASTERN CANCER STUDY GROUP) (1986). Consolidation and maintenance therapy in multiple myeloma: randomized comparison of a new approach to therapy after initial response to treatment. *J. Clin. Oncol.*, **4**, 888–899.

COOPER, M.R., MCINTYRE, O.R., PROPERT, K.J., KOCHWA, S., ANDERSON, K., COLEMAN, M., KYLE, R.A., PRAGER, D., RAFLA, S. & ZIMMER, B. (1986). Single, sequential, and multiple alkylating agent therapy for multiple myeloma: a CALGB study. *J. Clin. Oncol.*, **9**, 1331–1339.

DE BARBIERI, A., CHIAPPINO, G., DI VITTORIO, P., GOLFERINI, A., MAUGERI, M., MISTRETTA, A.P., PERRONE, F., TASSI, G.C., TEMELCOU, O. & ZAPPELLI, P. (1972). Peptichemio. A synthesis of pharmacological, morphological, biochemical and biomolecular investigations. In Proceedings of Symposium on Peptichemio, De Barbieri, A. (ed.) pp. 44–46. Istituto Sieroterapico Milanese: Milan.

DURIE, B.G.M. & SALMON, S.E. (1975). A clinical staging system for multiple myeloma. *Cancer*, **36**, 842–854.

GARTHON, B.M., TURA, S., LJUNGMAN, P., BELANGER, C., BRANDT, L., CAVO, M., FACON, T., GRANENA, A., GORE, M., GRATWOHL, A., LOWENBERG, B., NIKOSKELAINEN, J., REIFFERS, J.J., SAMSON, D., VERDONCK, L. & VOLIN, L. (FOR THE EUROPEAN GROUP FOR BONE MARROW TRANSPLANTATION) (1991). Allogeneic bone marrow transplantation in multiple myeloma. *N. Engl. J. Med.*, **325**, 1267–1273.

GREGORY, W.M., RICHARDS, M.A. & MALPAS, J.S. (1992). Combination chemotherapy versus melphalan and prednisone in the treatment of multiple myeloma: an overview of published trials. *J. Clin. Oncol.*, **10**, 334–342.

HARLEY, J.B., PAJAK, T.F., MCINTYRE, O.R., KOCHWA, S., COOPER, M.R., COLEMAN, M. & CUTTNER, J. (1979). Improved survival of increased-risk myeloma patients on combined triple-alkylating-agent therapy: a study of the CALGB. *Blood*, **54**, 12–22.

HJORTH, M., HELLQUIST, L., HOLMBERG, E., MAGNUSSON, B., RÖDIER, S. & WESTIN, J. (FOR THE MYELOMA GROUP OF WESTERN SWEDEN) (1990). Initial treatment in multiple myeloma: no advantage of multidrug chemotherapy over melphalan-prednisone. *Br. J. Haematol.*, **74**, 185-191.

HJORTH, M., HELLQUIST, L., HOLMBERG, E., MAGNUSSON, B., RÖDJER, S. & WESTIN, J. (1993). Initial versus deferred melphalan-prednisone therapy for asymtomatic multiple myeloma stage I: a randomized study. *Eur. J. Haematol.*, **50**, 95-102.

KILDAHL-ANDERSEN, O., BJARK, P., BONDEVIK, A., BULL, O., DEHELI, O., KVAMBE, V., NORDAHL, E., YTREHUS, K. & LAMVIK, J. (1988). Multiple myeloma in central and northern Norway 1981-82: a follow-up study of a randomized clinical trial of 5-drug combination therapy versus standard therapy. *Eur. J. Haematol.*, **41**, 47-51.

MACLENNAN, I.C.M. & CUSICK, J. (FOR THE MEDICAL RESEARCH COUNCIL WORKING PARTY ON LEUKAEMIA IN ADULTS) (1985). Objective evaluation of the role of vincristine in induction and maintenance therapy for myelomatosis. *Br. J. Cancer*, **52**, 153-158.

MACLENNAN, I.C.M., KELLY, K., CROCKSON, R.A., COOPER, E.H., CUZICK, J. & CHAPMAN, R.A. (1988). Results of the MRC myelomatosis trials for patients entered since 1980. *Hematol. Oncol.*, **6**, 145-158.

MACLENNAN, I.C.M., CHAPMAN, R.A., DUNN, J. & KELLY, K. (FOR THE MEDICAL RESEARCH COUNCIL WORKING PARTY FOR LEUKAEMIA IN ADULTS) (1992). Combined chemotherapy with ABCM versus melphalan for treatment of myelomatosis. *Lancet*, **339**, 200-205.

MERLINI, G.P., GOBBI, P.G., RICCARDI, A., RIVA, G., SARDI, C. & PERUGINI, S. (1982). Peptichemio induction therapy in myelomatosis. *Cancer Chemother. Pharmacol.*, **8**, 9-16.

MERLINI, G.P., RICCARDI, A., RICCARDI, P.G., MONTECUCCO, C.M., PAVESI, F. & ASCARI, E. (1985). Peptichemio, Vincristine, prednisone induction treatment in multiple myeloma. *Tumori*, **71**, 581-588.

MONCONDUIT, M., MENARD, J.F., MICHAUX, J.L., LE LOET, X., BERNARD, J.F., GROSBOIS, B., POLLET, J.P., AZAIS, I., LAPORTE, J.P., DOYEN, C., DE GRAMONT, A., WETTERWALD, M., DUCLOS, B., EULLER-ZIEGLER, L. & PENY, A.M. (FOR THE GROUPE D'ETUDES ET DE RECHERCHE SUR LE MYÉLOME) (1992). VAD or VMBCP in severe multiple myeloma. *Br. J. Haematol.*, **80**, 199-204.

MONTECUCCO, C.M., RICCARDI, A., MERLINI, G.P., MAZZINI, G., GIORDANO, P., DANOVA, M. & ASCARI, E. (1984). Plasma cell DNA content in multiple myeloma and related paraproteinemic disorders. Relationship with clinical and cytokinetic features. *Eur. J. Cancer Clin. Oncol.*, **20**, 81-89.

MONTECUCCO, C.M., RICCARDI, A., MERLINI, G.P. & ASCARI, E. (1986). Complete remission in plasma cell leukaemia. *Br. J. Haematol.*, **62**, 525-527.

PEEST, D., DEICHER, H., COLDEWEY, R., SCHMOLL, H.J. & SCHEDEL, I. (1988). Induction and maintenance therapy in multype myeloma: a multicenter trial of MP versus VCMP. *Eur. J. Cancer Clin. Oncol.*, **24**, 1061-1067.

POMMATAU, E., MATHE', G. & HAYAT, M. (FOR THE EUROPEAN ORGANIZATION ON RESEARCH ON TREATMENT OF CANCER, EORTC) (1977). A phase II clinical trial of Peptichemio. *Biomedicine*, **27**, 290-294.

RICCARDI, A., MARTINOTTI, A., & PERUGINI, S. (1978). Cytokinetic changes in two cases of plasma cell leukemia treated with a multipeptide derivative of m-{di(2-chloroethyl)amino}-L-phenylalanine (Peptichemio). *Eur. J. Cancer*, **14**, 1099-1104.

RICCARDI, A., MONTECUCCO, C.M., MERLINI, G.P., UCCI, G., CREMONINI, N., GOBBI, P.G. & ASCARI, E. (1984). Proliferative activity, response to therapy and survival in multiple myeloma. *Haematologica*, **69**, 148-162.

RICCARDI, A., MONTECUCCO, C.M., DANOVA, M., UCCI, G., MERLINI, G.P. & ASCARI, E. (1985). Rate of M-component changes and plasma cell labelling index in 25 patients with multiple myeloma treated with Peptichemio. *Cancer Treat. Rep.*, **69**, 971-975.

RICCARDI, A., MERLINI, G.P., MONTECUCCO, C.M., DANOVA, M., UCCI, G., CASSANO, E. & ASCARI, E. (1986). Peptichemio, Vincristine and prednisone versus melphalan and prednisone as induction therapy in multiple myeloma. *Eur. J. Cancer Clin. Oncol.*, **22**, 787-791.

RICCARDI, A., GIORDANO, M. & GIRINO, M. (1987). Treatment of acute non-lymphoblastic leukemia: a computer-aided analysis. *Haematologica*, **72**, 71-88.

RICCARDI, A., UCCI, G., LUONI, R., CASTELLO, A., COCI, A., MAGRINI, U. & ASCARI, E. (FOR THE COOPERATIVE GROUP OF STUDY AND TREATMENT OF MULTIPLE MYELOMA) (1990). Bone marrow biopsy in monoclonal gammopathies: correlations between pathologic findings and clinical data. *J. Clin. Pathol.*, **43**, 469-475.

RICCARDI, A., GOBBI, P.G., UCCI, G., BERTOLONI, D., LUONI, R., RUTIGLIANO, L. (1991). Changing clinical presentation of multiple myeloma. *Eur. J. Cancer*, **27**, 1401-1405.

SALMON, S.E., HAUT, A., BONNET, J.D., AMARE, M., WEICK, J.K., DURIE, B.G.M. & DIXON, D.O. (1983). Alternating combination chemotherapy and levamisole improves survival in multiple myeloma. A Southwest Oncology Group Study. *J. Clin. Oncol.*, **1**, 453-461.

UCCI, G., PETRINI, M., RICCARDI, A., INVERNIZZI, R., CARULLI, G., LUONI, R., GIORDANO, M. & DANOVA, M. (1992). Expression of p170 protein in multiple myeloma: a clinical study. *Hematol. Oncol.*, **10**, 213-220.